# ASO Author Reflections: Distal Pancreatectomy with Celiac Axis Resection for Locally Advanced Pancreatic Cancer—Patient Selection and Surgical Experience are Key

**DOI:** 10.1245/s10434-018-7033-1

**Published:** 2018-11-19

**Authors:** Sjors Klompmaker, Marc G. Besselink

**Affiliations:** Department of Surgery, Cancer Center Amsterdam, Amsterdam UMC, University of Amsterdam, Amsterdam, The Netherlands

## Past

In contrast to most other cancers, the 5-year survival rate of pancreatic cancer has only improved marginally, from 2.5% to 8.2%, in the past 40 years.^1^ As a result, pancreatic cancer is projected to become the second-leading cause of cancer deaths by 2030.^2^ Surgical resection improves survival, but only 10–20% of patients are deemed upfront resectable.^3^ A select group of patients with locally advanced pancreatic cancer involving the celiac axis, not upfront resectable according to most guidelines, could benefit from a distal pancreatectomy with celiac axis resection (DP-CAR).^4^ However, evidence on indications and outcome of DP-CAR was scarce and multicenter cohort studies from Western countries were lacking. This pan-European multicenter study addressed the short-term and oncologic outcomes of this procedure.^5^

## Present

The study included 68 patients undergoing DP-CAR at 20 hospitals across 12 European countries, between 2000 and 2016. The 90-day mortality rate was 16%, and among the 62 patients treated for pancreatic ductal adenocarcinoma, median overall survival was 18 (95% confidence interval 10–37) months. The majority of patients (82%) also had received chemotherapy and/or radiotherapy. The study found that 7 of the 11 patients with 90-day mortality died of ischemia-related causes. Preoperative embolization of the hepatic artery was not significantly associated with fewer ischemic complications. The median hospital case volume was only three per year. No association between institutional volume and mortality was found, potentially due to the relatively small sample size.

## Future

First and foremost, future research should focus on reducing 90-day mortality after DP-CAR, because the overall rate of 16% seems to high to justify the modest survival benefit. Four strategies could offer improvement. First, better selection of patients may improve outcomes. For instance, by selecting fitter patients who do not require extensive additional vascular or organ resection. Second, limiting this procedure to centers who regularly perform DP-CAR may reduce surgical complications and improve postoperative management. Third, selecting patients with stable disease after 2–4 months of treatment with induction (FOLFIRINOX) chemotherapy may improve overall survival and avoid futile resections for aggressive cancer subtypes. Finally, translational research could focus on strategies to reduce the physiological impact and postoperative ischemia caused by resection of the celiac axis. For the immediate future, we expect to see improved short-term mortality and survival when the DP-CAR is performed on well-selected patients at experienced high-volume centers.
